# LncRNA GACAT3 promotes esophageal squamous cell carcinoma progression through regulation of miR-149/FOXM1

**DOI:** 10.1186/s12935-021-02192-4

**Published:** 2021-09-08

**Authors:** Min Su, Jinming Tang, Baihua Zhang, Desong Yang, Zhining Wu, Jie Wu, Yong Zhou, Qianjin Liao, Hui Wang, Wenxiang Wang, Yuhang Xiao

**Affiliations:** 1 Hunan Clinical Medical Research Center of Accurate Diagnosis and Treatment for Esophageal carcinoma, Hunan Cancer Hospital and The Affiliated Cancer Hospital of Xiangya School of Medicine, Central South University, Changsha, 410013 Hunan People’s Republic of China; 2 Thoracic Surgery Department 2, Hunan Cancer Hospital and The Affiliated Cancer Hospital of Xiangya School of Medicine, Central South University, Changsha, 410013 Hunan People’s Republic of China; 3 Hunan Key Laboratory of Cancer Metabolism, Hunan Cancer Hospital and The Affiliated Cancer Hospital of Xiangya School of Medicine, Central South University, Changsha, 410013 Hunan People’s Republic of China; 4 Hunan Key Laboratory of Translational Radiation Oncology, Hunan Cancer Hospital and The Affiliated Cancer Hospital of Xiangya School of Medicine, Central South University, Changsha, 410013 Hunan People’s Republic of China; 5 Department of Pharmacy, Xiangya Hospital of Xiangya School of Medicine, Central South University, Changsha, 410001 Hunan People’s Republic of China

**Keywords:** Esophageal squamous cell carcinoma, Long noncoding RNA, GACAT3, miR-149, FOXM1

## Abstract

**Background:**

The long noncoding RNA gastric cancer associated transcript 3 (GACAT3) has been demonstrated to be implicated in the carcinogenesis and progression of many malignancies. However, GACAT3’s levels and role in esophageal squamous cell carcinoma (ESCC) has not been elucidated.

**Methods:**

GACAT3 amounts were investigated in ESCC tissues and cell lines by qPCR. Its biological functions were examined by CCK-8 assay, colony formation assay, flow cytometry, wound healing assay, transwell assay, and xenograft model establishment. The relationship between GACAT3 and miR-149 was assessed by dual-luciferase reporter assay.

**Results:**

GACAT3 amounts were elevated in ESCC tissue and cell specimens. Functional studies showed that GACAT3 silencing reduced the proliferation, migration and invasion of cultured ESCC cells, and decreased tumor growth in mice. Furthermore, GACAT could directly interact with miR-149. In addition, colony formation and invasion assays verified that GACAT3 promotes ESCC tumor progression through miR-149. Moreover, GACAT3 acted as a competing endogenous RNA (ceRNA) to modulate FOXM1 expression.

**Conclusions:**

These findings indicate that GACAT3 functions as an oncogene by acting as a ceRNA for miR-149 to modulate FOXM1 expression in ESCC, suggesting that GACAT3 might constitute a therapeutic target in ESCC.

## Introduction

Esophageal cancer (EC), the ninth commonest malignancy, is the sixth deadliest cancer globally [1, 2]. ECs can be broadly divided into esophageal adenocarcinoma and esophageal squamous cell carcinoma (ESCC). Although clinical interventions have been recently improved, EC prognosis is disappointing with 5-year survival of less than 20% [3]. This poor survival rate of EC patients is due to late diagnosis, frequent metastasis and rapid progress of tumor [4]. In addition, the precise genetics and molecular mechanisms of ESCC are undefined [3]. Therefore, there is a dire need to develop new diagnostic and prognostic markers to identify patients at the early stage, as well as target genes associated with the progression of ESCC, to improve the survival of patients.

Recent whole-genome sequencing data suggested the active transcription of more than 90% of the human genome [5]. However, only 2% of all transcripts are translated into proteins, with the majority being non-coding RNAs (ncRNAs) [6]. Of these ncRNAs, long ncRNAs (lncRNAs) attract increasing attention. LncRNAs are an important class of transcripts exceeding 200 nucleotides, showing no or low protein-coding potential [7, 8]. Mounting evidence suggests lncRNAs commonly exhibit dysregulated expression in cancer and may emerge as essential regulators, with essential functions in carcinogenesis and tumor progression [9, 10].

LncRNA gastric cancer associated transcript 3 (GACAT3), also termed AC130710, is located on human Chr2p24.3 [11]. GACAT3 was originally identified as a novel lncRNA based on a lncRNA microarray analysis of gastric cancer tissues [12]. Another report demonstrated GACAT3 is overexpressed in GC tissues and contributes to GC progression [13]. Subsequently, further studies have demonstrated that *GACAT3* acts as an oncogenic lncRNA and contributes to the development of various cancers, including breast cancer [11], colorectal cancer [14], bladder cancer [15], and glioma [16]. These findings indicate *GACAT3* may play critical roles in multiple cancers, constituting a potential molecular marker. However, GACAT3’s function and potential regulatory roles in ESCC remain undefined. Therefore, the present work aimed to assess GACAT3’s levels, function and mechanisms in EC.

## Methods

### Clinical specimens and cells

Totally 46 clinical ESCC tissue and adjacent non-tumorous tissue specimens were collected perioperatively at the Hunan Cancer Hospital of Central South University (Changsha, China). None of these patients had been pretreated with chemotherapy or radiotherapy prior to the surgery. Histopathological diagnosis was confirmed by experienced pathologists. The specimens were snap frozen in liquid nitrogen and kept at –80 °C. This study had approval from the ethics committee of the Hunan Cancer Hospital. Each patient provided written informed consent.

Human ESCC KYSE-150 and KYSE-510 cells, and human normal esophageal epithelial HET-1 A cells were provided by the Institute of Cell Research, Chinese Academy of Sciences (Shanghai, China). All cells were maintained in RPMI 1640 containing 10% fetal bovine serum (FBS) and 100 U/ml penicillin and streptomycin, in a humid 5% CO_2_ incubator at 37 °C.

### Quantitative reverse transcription polymerase chain reaction (qRT-PCR)

Total RNA was extracted from tissue and cell specimens with TRIzol (Invitrogen, USA) as directed by the manufacturer. A SYBR Green PCR Kit (Takara, Japan) was utilized to detect lncRNAs and mRNAs. GAPDH was utilized to normalize transcript levels. MicroRNAs were assessed with a miDETECT A Track Kit (RiboBio, China), using U6 as a reference. The relative quantification of RNAs was performed by the 2^−△△Ct^ method. The sequences of the primers used in the study were listed in Table 1.

**Table 1:** QRT-PCR primer sequences

Gene	Forward	Reverse
GACAT3	5′-CTTCCGGAGCAGGTCTGAGT-3′	5′-CTTTCCCTGCAGAGACCAGT-3′
FoxM1	5′-TATTCACAGCATCATCACAGC-3′	5′-GAAGGCTCCTCAACCTTAACCT-3′
miR-149-5p	5′-GGCTCTGGCTCCGTGTCTT-3′	5′-CAGTGCAGGGTCCGAGGTATT-3′

### Immunoblot

Total protein extraction from cells utilized the RIPA buffer (Beyotime, China) containing protease inhibitors (Roche, Switzerland). Immunoblot was performed as previously described [17]. Anti-rabbit antibodies against FOXM1 (1:500, 4 A Biotech, China) were used as primary antibodies. Anti-rabbit GAPDH antibodies (1:5000, ZENbio) were utilized as a reference. HRP-linked IgG (1:5000, Beyotime China) was employed as secondary antibodies. Blots were developed with the ECL-Plus reagent (Millipore, USA).

### Transfection

shRNAs against the target GACAT3 and the corresponding negative control shRNA were introduced in a lentiviral vector (GeneChem, China) and used to transfect ESCC cells. This was followed by a 7-day incubation with 2 µg/ml puromycin for selecting stable transfectants. GACAT3 expression was then assessed by qRT-PCR.

### CCK-8 assay

CCK-8 was utilized to measure the viability of ESCC cells, as directed by the manufacturer. Briefly, the cells seeded in 96-well plates at 2 × 10^3^/well were assessed for proliferation daily for 4 days. 10 µl of CCK-8 solution/well was added for 1 h at 37 °C, and optical density was obtained at 450 nm spectrophotometrically.

For the colony formation assay, 1 × 10^3^ cells were seeded into a 35-mm dish and incubated for 2 weeks under routine conditions. This was followed by 4% paraformaldehyde fixation (15 min) and 0.1% crystal violet staining. Images of colonies (> 50 cells) were taken under a light microscope, and the number of colonies was analyzed by the ImageJ software.

### 5-ethynyl-2’- deoxyuridine (EdU) experiment

EdU assay was conducted using EdU Cell Proliferation Kit with Alexa Fluor 555 (Epizyme, China). In short, cells were plated into 24-well plates and cultivated overnight. Following treatment with EdU reagent for 2 h at 37 °C, cells were fixed with 4% paraformaldehyde and mixed with 0.5% Triton-X-100 (Sigma-Aldrich). Nuclei were dyed with Hoechst 33,342. The images were captured with a fluorescence microscope (Olympus, Japan, ×200).

### Cell cycle analysis

Cells plated into 6-well plates underwent a 24-h culture, then collected cells were fixed overnight at 4 °C in 70% ethanol. The liquid was discarded after centrifugation, and cells were stained with PI and RNase solution form Cell Cycle and Apoptosis Analysis Kit (Beyotime, China). Samples were incubated for 0.5 h at 37 °C, and then the cell cycle status were measured by FACS Calibur flow cytometer (BD Biosciences).

### In vitro migration and invasion assays

Cells in 6-well plates were grown to 80–90% confluence. Then, the monolayer was scratched using a sterile pipette tip, followed by culture in medium containing 1% FBS. At 0 and 24 h, respectively, cells were imaged under an Olympus microscope (×100).

The transwell assay was carried out for measuring cell invasion by using 24-well chambers with 8-µm pore membranes (Corning, USA). After resuspension in 200 µl serum-free medium at 1 × 10^6^/ml, cells were placed in the Matrigel (BD Biosciences)-coated upper chamber of the transwell plate. Meanwhile, 600 µl of medium with 20% FBS was added to the lower chamber. After 24 h of incubation, invasive cells (lower surface of the insert) underwent 0.1% Crystal Violet staining. A microscope was utilized to count the invasive cells (×100) in 5 randomly selected high-power fields per specimen.

### Apoptosis assessment

Apoptosis evaluation utilized the Annexin-VFITC apoptosis detection kit (BD, USA) as directed by the manufacturer. Cells in 6-well plates underwent a 24 h culture in normal medium, trypsinization and resuspension in 100 µl binding buffer. This was followed by staining with 5 µl Annexin V/FITC and 5 µl PI at ambient for 15 min shielded from light. A FACS Calibur flow cytometer (BD Biosciences) was used for analysis.

### Luciferase reporter assay

Bioinformatics tools (microRNA.org) were utilized for predicting miR-149 binding of GACAT3. Human 293T cells underwent co-transfection with 160 ng of empty pmirGLO-GACAT3-wt or pmirGLO-GACAT3-mut and 5 pmol miR-149 mimic or miR-NC in presence of Lipofectamine 2000 (Invitrogen) as directed by the manufacturer. Luciferase assay was carried out after 48 h of incubation, with the Dual Luciferase Reporter Assay System (Promega, USA).

### Tumor xenograft assays

Female BALB/c nude mice (4 weeks old) underwent housing under specific pathogen-free conditions. For establishing the xenograft model, KYSE-510 cells stably transfected with sh-GACAT3 or sh-NC (5 × 10^6^/0.2 ml PBS supplemented with 10% Matrigel) were subcutaneously implanted into the animal’s armpit. Tumor sizes were monitored at 3-day intervals using digital calipers, to determine tumor volumes as follows: volume = 1/2 (length × width^2^). After 4 weeks, the mice were placed in a clear, clean cage, which measuring 28 cm × 20 cm × 18 cm (volume = 10.08 l). Then, CO_2_ were introduced into the cage at 2 l per min. After mice breathing stops (approximately 3 min), cervical dislocation was performed. Then, tumors were extracted and weighed, tumor specimens underwent paraffin embedding and hematoxylin and eosin (H&E) or immunohistochemical staining. The Guidelines for the Care and Use of Laboratory Animals were strictly followed in this study approved by the Ethics Committee of Hunan cancer hospital.

### Statistical analysis

SPSS 18.0 (SPSS, USA) and GraphPad Prism 6 (GraphPad, USA) were utilized for data analysis. Data are mean ± SD and were compared by Student’s t test (group pairs). Experiments were repeated three times or more. P < 0.05 indicated statistical significance.

## Results

### GACAT3 is upregulated in ESCC

**Fig. 1:**
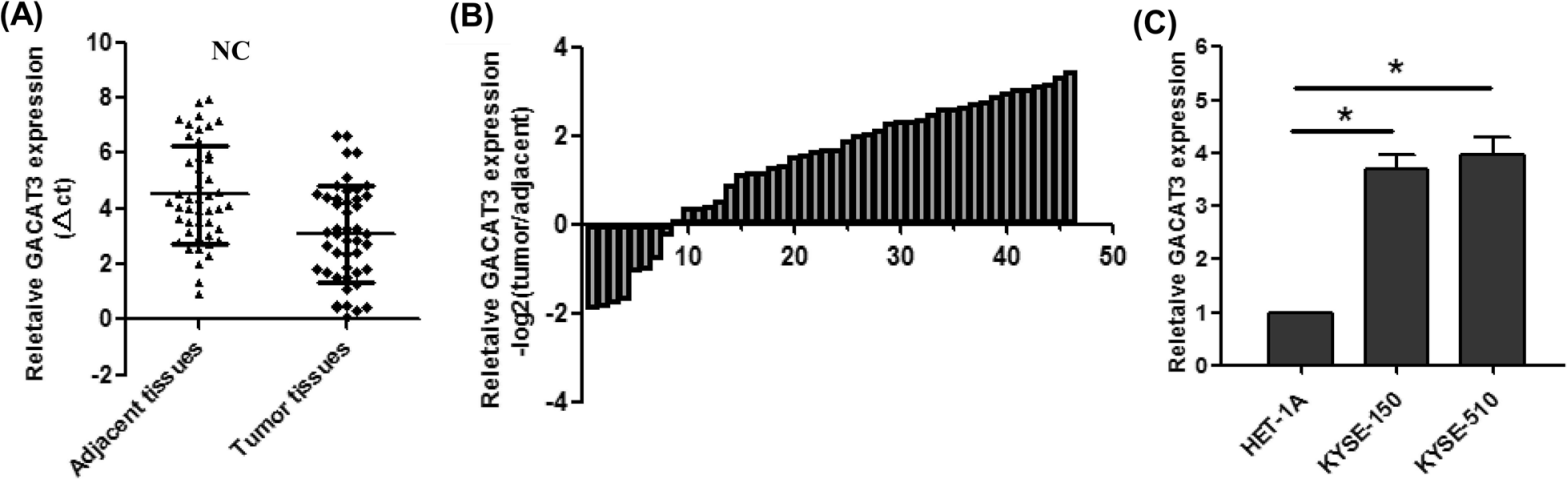
GACAT3 is overexpressed in ESCC tissue samples and cells. **A**–**B** Relative expression levels of GACAT3 in ESCC and adjacent noncancerous tissue samples assessed by qRT-PCR (n = 46). **C** Relative expression levels of GACAT3 in human ESCC and esophageal epithelial HET-1 A cells. *P < 0.05, **P < 0.01, ***P < 0.001

We first analyzed the expression levels of GACAT3 in ESCC and adjacent noncancerous tissue specimens. qRT-PCR demonstrated GACAT3 overexpression in ESCC tissue samples (Fig. 1 A and B). In agreement, GACAT3 was upregulated in human ESCC KYSE-150 and KYSE-510 cells in comparison with human esophageal epithelial HET-1 A cells (Fig. 1C).

### GACAT3 mediates ESCC cell proliferation and apoptosis in vitro

**Fig. 2:**
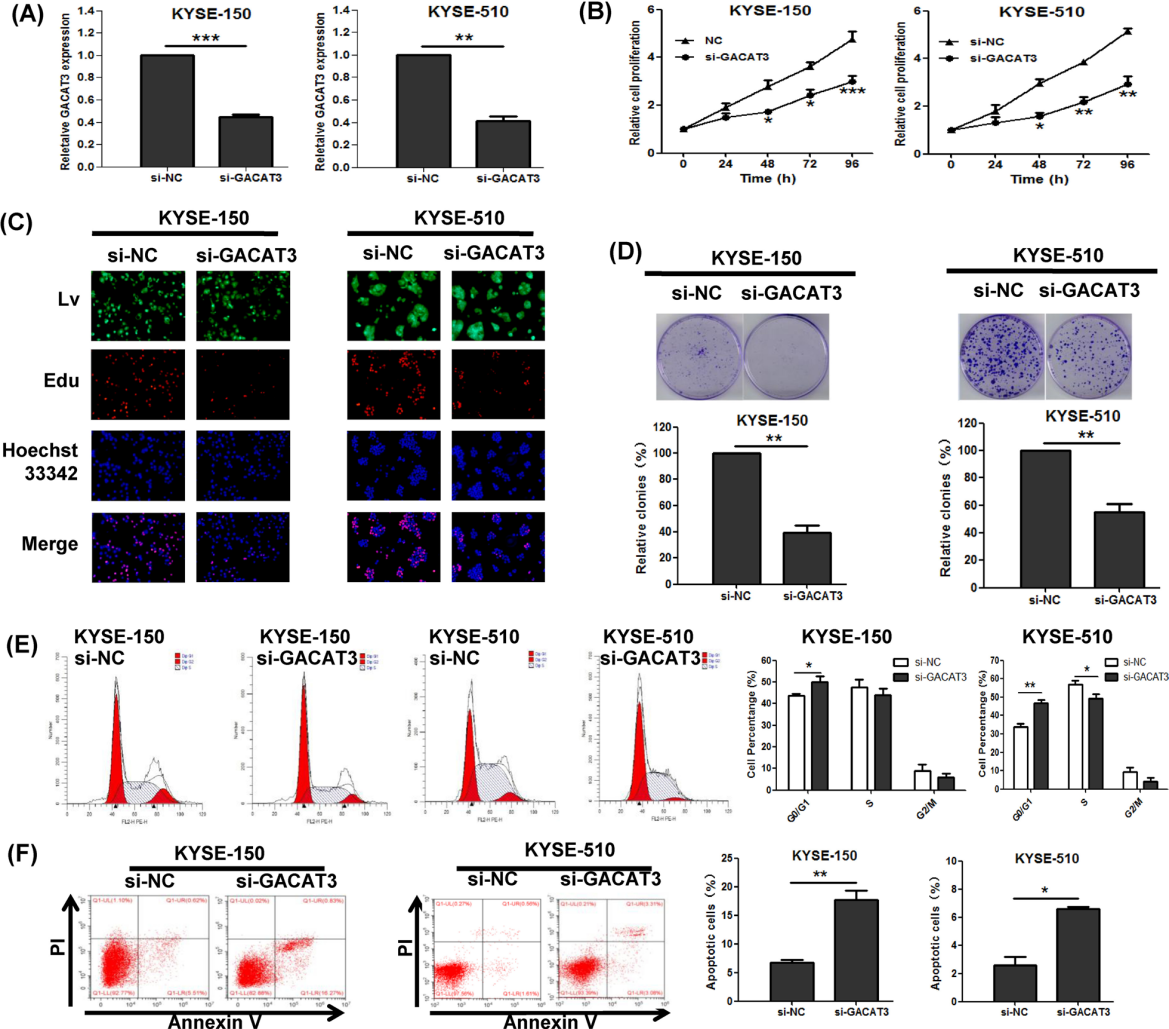
GACAT3 controls cell proliferation and apoptosis in cultured ESCC cells. **A** GACAT3 expression was knocked down by shRNA targeting GACAT3 in KYSE-150 and KYSE-510 cells. Effects of GACAT3 on cell proliferation, examined by (**B**) CCK-8, **C** Edu and **D** colony formation assays. **E** Cell cycle and **F** apoptotic rates of ESCC cells after transfection with GACAT3, examined by FACS analysis. *P < 0.05, **P < 0.01, ***P < 0.001

To further elucidate the biological significance of GACAT3 in ESCC progression, cell proliferation and apoptosis were examined. We first designed small hairpin RNA (shRNA) targeting GACAT3 and showed that it could knockdown GACAT3 in KYSE-150 and KYSE-510 cells (Fig. 2A). Then, CCK-8 and Edu assays showed GACAT3 silencing reduced cell proliferation (Fig. 2B, C). Additionally, markedly reduced clonogenic survival were obtained following GACAT3 silencing (Fig. 2D). Subsequently, flow cytometry was used to examine cell cycle distribution in KYSE-150 and KYSE-510 cells, which indicated that GACAT3 silencing increased the proportion of G0/G1 cells and decreased the number of S phase cells (Fig. 2E). Meanwhile, apoptotic cells were increased after transfection with sh-GACAT3, as assessed by FACS analysis (Fig. 2F). These data indicated that GACAT3 induced proliferation, and suppressed apoptosis in cultured ESCC cells.

### GACAT3 mediates EC cell migration and invasion in vitro

**Fig. 3:**
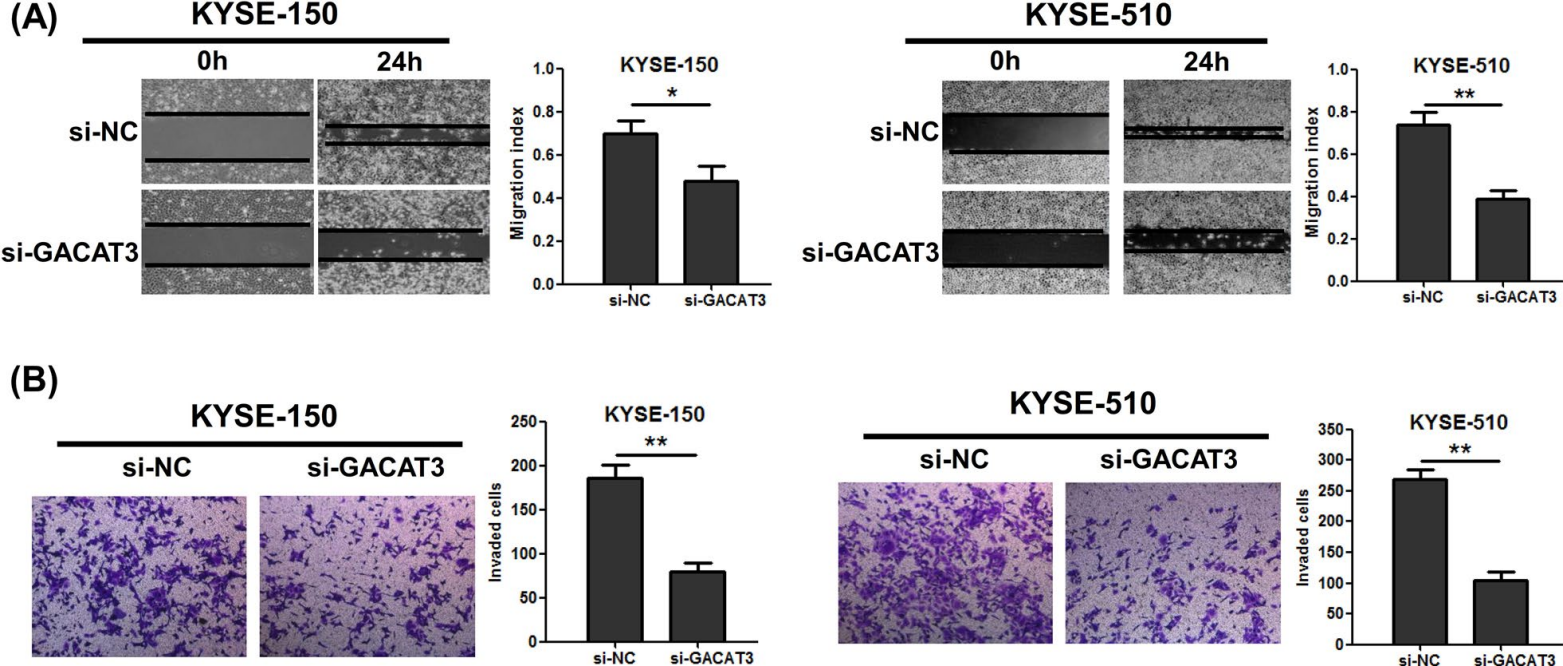
GACAT3 modulates cell migration and invasion in cultured ESCC cells. **A** Effect of GACAT3 on cell migration in the wound healing assay. **B** Effect of GACAT3 on cell invasion in the Matrigel transwell assay. *P < 0.05, **P < 0.01, ***P < 0.001

Next, we assessed the migratory and invasive abilities of cells following GACAT3 knockdown. As shown in Fig. 3A, the wound-healing assays showed that suppressing GACAT3 expression in KYSE-150 and KYSE-510 underwent slower scratch wound closure than the negative control cells. Matrigel transwell assays revealed that GACAT3 knockdown inhibited invasion abilities of ESCC cells (Fig. 3B). Jointly, the above findings suggested GACAT3 markedly promoted migration and invasion in ESCC cells.

### GACAT3 interacts with and downregulates miR-149

**Fig. 4:**
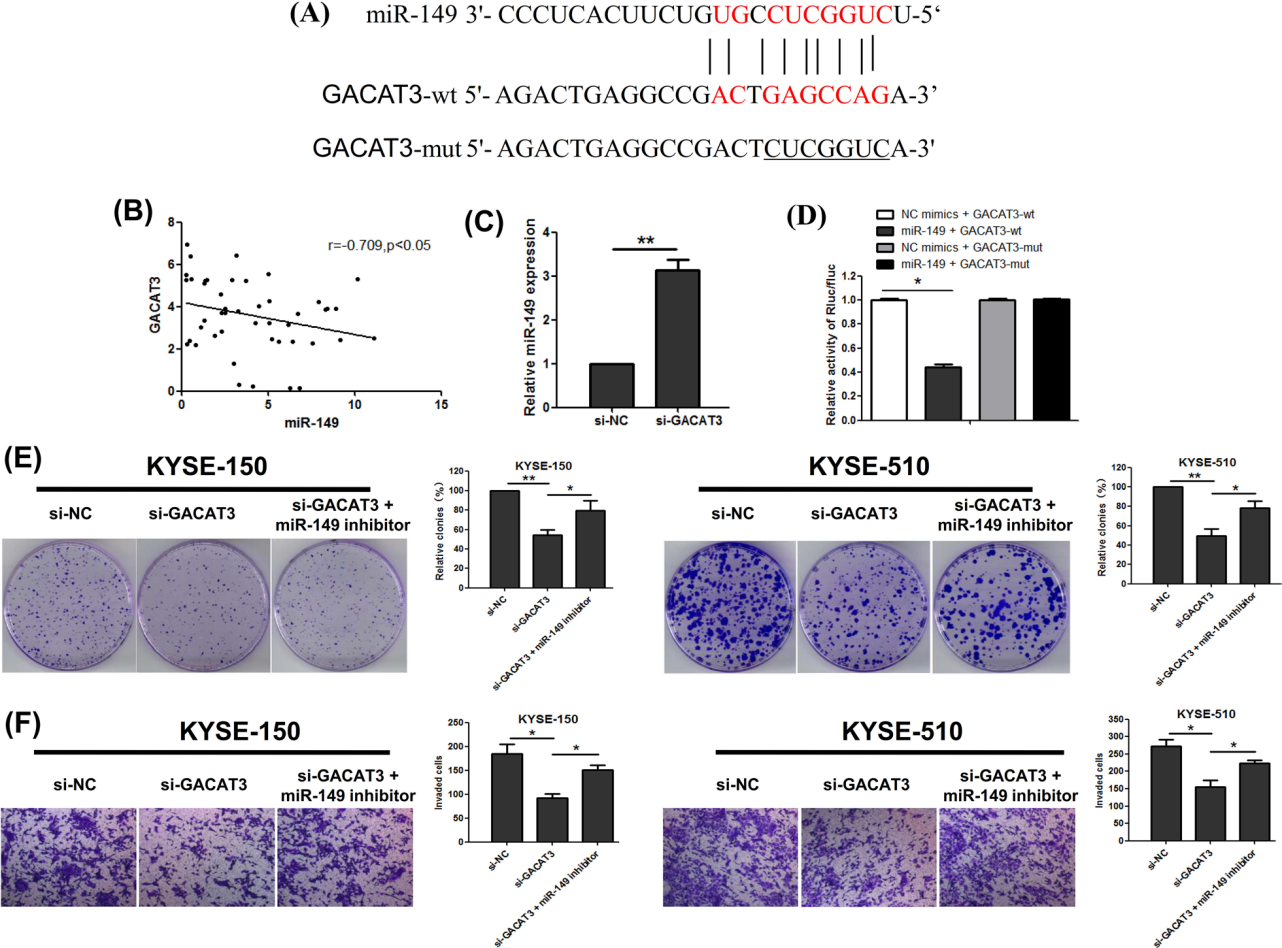
GACAT3 acts as a sponge of miR-149 in ESCC cells. **A** Sequence alignment of miR-149 with binding sites in the wild-type and mutant-type regions of GACAT3. **B** Negative correlation between miR-149 and GACAT3, determined by Pearson’s correlation analysis. **C** Relative luciferase activity in 293T cells was assessed after co-transfection with the reporter plasmid (GACAT3-wt and GACAT3-mut, respectively) and miRNAs (miR-149 and NC mimics, respectively). **D** miR-149 levels in KYSE-510 cells transfected with sh-GACAT3, assessed by qRT-PCR. Attenuated effects of miR-149 inhibitor on reduced (**E**) cell proliferation and **F** invasion ability related to sh-GACAT3 examined by colony formation and Matrigel transwell assays. *P < 0.05, **P < 0.01, ***P < 0.001

Recently, a lot of lncRNAs have been revealed to function as competing endogenous RNAs (ceRNA) by competitively binding miRNAs. To explore the targeting of GACAT3, we first analyzed the potential target miRNAs by bioinformatics analysis. Starbase v2.0 were employed for predicting putative miRNA binding sites in GACAT3. The results revealed miR-149 as a GACAT3 target (Fig. 4A). MiR-149 levels were remarkably reduced in ESCC tissue samples compared with adjacent tissue specimens. Pearson correlation analysis indicated GACAT3 and miR-149 were negatively correlated in ESCC tissue samples (Fig. 4B). In addition, qRT-PCR showed that knockdown of GACAT3 significantly increased miR-149 expression in KYSE-510 cells (Fig. 4C).

To confirm the direct binding between GACAT3 and miR-149, a GACAT3-mt luciferase reporter vector was generated, with mutations in the predicted miR-149 binding sites. Dual luciferase reporter assays revealed luciferase activity of wt-GACAT3 was starkly decreased by miR-149 mimic (Fig. 4D), while mut-GACAT3 caused no significant changes. Besides, the inhibition of miR-149 in GACAT3-knockdown KYSE-150 and KYSE-510 cells reversed the decrease of proliferation and invasion ability (Fig. 4E, F). The above data suggested an interaction between miR-149 and GACAT3.

### GACAT3 acts as a ceRNA in FOXM1 regulation by sponging miR-149

**Fig. 5:**
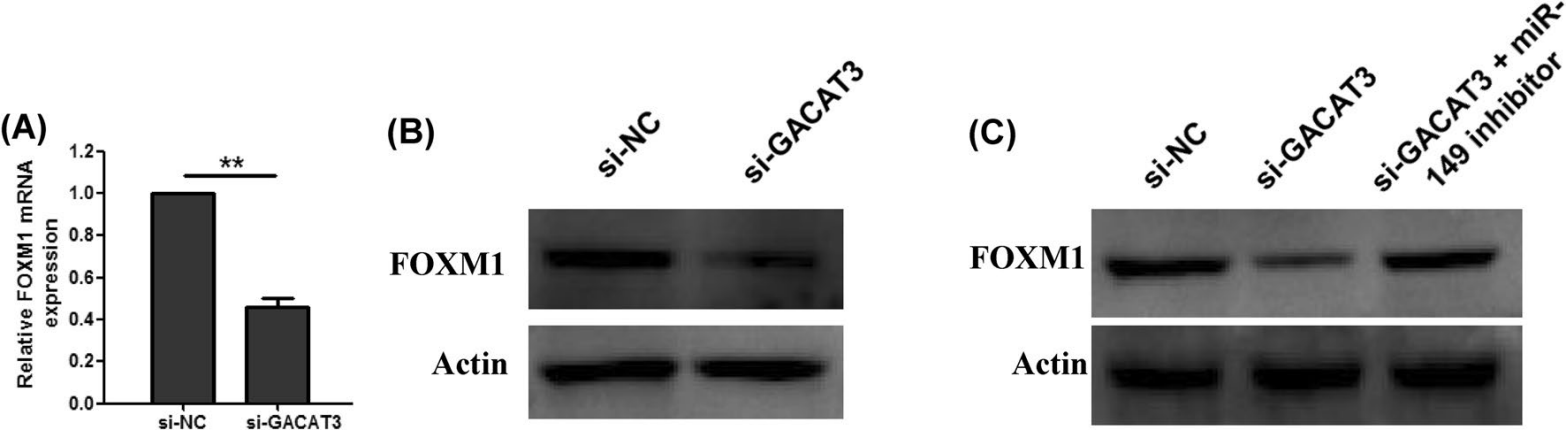
GACAT3 regulates FOXM1 through miR-149 in ESCC cells. **A** FOXM1 mRNA levels in KYSE-510 cells transfected with sh-GACAT3, assessed by qRT-PCR. **B** FOXM1 protein levels in KYSE-510 cells transfected with sh-GACAT3, assessed by immunoblot. **C** FOXM1 protein levels in KYSE-510 cells co-transfected with sh-GACAT3 and miR-149 inhibitor, measured by immunoblot. *P < 0.05, **P < 0.01, ***P < 0.001

The miRNAs are known to bind their targets and cause RNA degradation and/or translational repression. A literature search revealed that FOXM1 is a miR-149 target, indicating that FOXM1 might contribute to the tumor-inducing function of GACAT3. To validate whether GACAT3 could regulate FOXM1 through miR-149, qRT-PCR and immunoblot assays were conducted. The results demonstrated that FOXM1 mRNA and protein amounts were reduced following GACAT3 silencing in KYSE-510 cells (Fig. 5A and B). Besides, inhibition of miR-149 could reverse FOXM1 protein level reduction associated with GACAT3 knockdown (Fig. 5C). Jointly, the above findings indicated GACAT3 regulated FOXM1 through sponging miR-149 in ESCC cells.

### GACAT3 promotes growth of ESCC cells in vivo

**Fig. 6:**
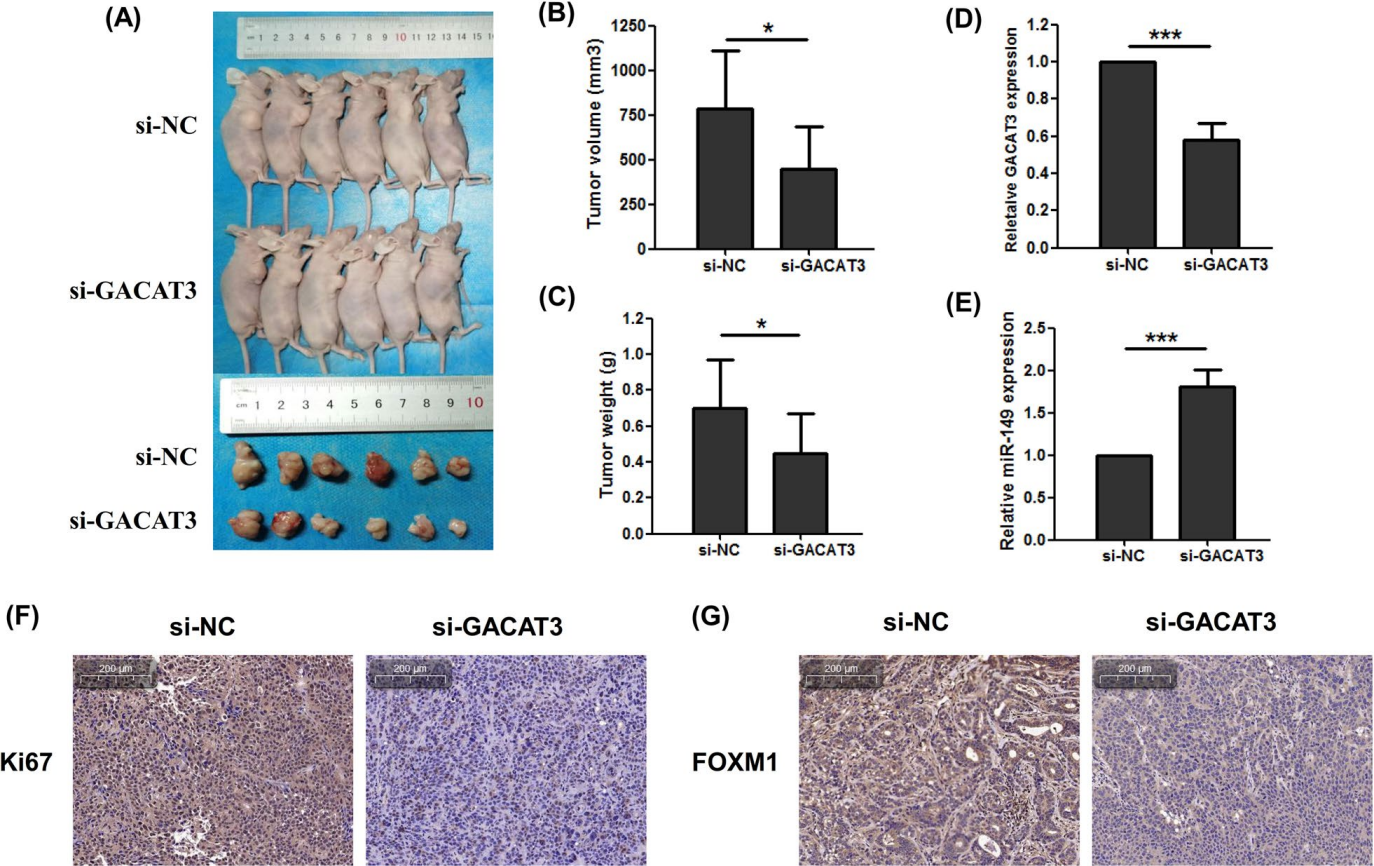
GACAT3 mediates ESCCC growth in vivo. **A** Representative images of tumors formed in nude mice injected with KYSE-510 cells transfected with sh-GACAT3 or sh-NC. **B** Tumor volumes of the xenograft tumors. **C** Tumor weights of the xenograft tumors. **D** GACAT3 and **E** miR-149 expression in xenograft tumor samples, detected by qRT-PCR. **F** Ki-67 and **G** FOXM1 expression in xenograft tumors, detected by immunohistochemistry (magnification, ×50; Scale bar, 200 μm.). *P < 0.05, **P < 0.01, ***P < 0.001

Next, GACAT3’s function was examined in vivo. To this end, KYSE-510 cells after stable transfection with sh-GACAT3 or control shRNA were subcutaneously implanted into BALB/c nude mice for establishing a xenograft model. As expected, tumor volumes and weights were starkly reduced in the sh-GACAT3 group in comparison with control values (Fig. 6A–C). In addition, tumor samples from the GACAT3 knockdown group showed decreased GACAT3 and increased miR-149 amounts (Fig. 6D and E). Furthermore, IHC demonstrated the xenografts in the GACAT3 knockdown group had lower Ki67 and FOXM1 expression than the group infected with control cells (Fig. 6F and G). Jointly, the above findings indicated GACAT3 markedly induced tumor growth in ESCC in vivo.

## Discussion

Recent evidence suggest that a variety of lncRNAs play critical roles in EC progression [10]. The current work revealed GACAT3 upregulation in ESCC tissue specimens and cells. Then, loss-of-function analyses suggested GACAT3 silencing suppressed cell growth, migration and invasion, induced cell cycle arrest in G0/G1 phase, and promoted apoptosis in cultured ESCC cells. Next, xenograft assays demonstrated GACAT3 knockdown reduced ESCC tumor growth in nude mice. The above findings indicate that GACAT3 might be an oncogene in ESCC.

In terms of mechanism, a major functional model proposes that lncRNAs act as ceRNAs for miRNA sponging through sequence complementarity, subsequently affecting miRNA functions [18]. MicroRNAs (miRNAs) are small ncRNAs of 17–24 nucleotides regulating genes post-transcriptionally via binding to the target mRNAs’ 3’-untranslated regions (3’-UTRs), causing translational inhibition and/or mRNA degradation [19]. Previous reports demonstrated GACAT3 might sponge miRNAs. For instance, Pan et al. reported that GACAT3 regulates glioma via miR-3127-5p [16]. Zhong et al. revealed that GACAT3 promotes breast cancer progression through miR-497 regulation [11]. Thus, we focused on miR-149 (one of the potential targets of GACAT3 predicted by bioinformatics software) and investigated whether it was regulated by GACAT3. MiR-149 is located at 2q37.3, encoded by one exon and found to have polymorphisms [20]. It is known that miR-149 is a tumor suppressor in various malignancies, with critical roles in cell proliferation, apoptosis, metastasis, chemoresistance and carcinogenesis regulation in humans [21, 22]. Moreover, miR-149 is downregulated in ESCC tissues, functioning as a tumor suppressor [23]. After miR-149 inhibition, ESCC cell proliferation and metastasis were remarkably enhanced [23]. As shown above, GACAT3 knockdown starkly upregulated miR-149 in ESCC cells. In agreement, a negative correlation between miR-149 and GACAT3 was observed in ESCC tissues. The subsequent luciferase reporter assay showed GACAT3 sponged miR-149 by directly interacting with its complementary sequence. These findings suggest GACAT3 plays an oncogenic role in ESCC by downregulating miR-149.

Forkhead box protein M1 (FoxM1) belongs to the family of Forkhead transcription factors that control G2/M progression and plays an important role in mitosis and chromosome segregation [24, 25]. FOXM1 is abnormally overexpressed in multiple cancers, causing abnormal functioning of malignant cells [26]. FOXM1 upregulation was also observed in ESCC and may indicate poor prognosis in stage IIA ESCC [27]. Silencing of FOXM1 suppressed proliferation and migration in ESCC cells, as shown above [28]. Moreover, several studies have reported FOXM1 as a direct miR-149 target [29, 30]. In this study, GACAT3 positively regulated FOXM1 in ESCC cells. More importantly, inhibition of miR-149 reversed GACAT3 knockdown-induced FOXM1 downregulation, suggesting GACAT3 could regulate FOXM1 expression by sponging miR-149.

## Conclusions

Overall, GACAT3 is upregulated in ESCC and acts as an oncogenic lncRNA in ESCC progression. Mechanistically, GACAT3 functions as a ceRNA by sponging miR-149 and subsequently promoting FOXM1 expression. Thus, GACAT3 should be considered a novel potential therapeutic target in ESCC.

## Data Availability

All the data involved in this study had been included in the manuscript, and the corresponding raw data could be acquired from the corresponding author upon reasonable request.

## Abbreviations

GACAT3: Gastric cancer associated transcript 3
ceRNA: Competing endogenous RNA
EC: Esophageal cancer
ESCC: Esophageal squamous cell carcinoma
FoxM1: Forkhead box protein M1
lncRNA: Long noncoding RNA
